# Comparing five depression measures in depressed Chinese patients using item response theory: an examination of item properties, measurement precision and score comparability

**DOI:** 10.1186/s12955-017-0631-y

**Published:** 2017-04-04

**Authors:** Yue Zhao, Wai Chan, Barbara Chuen Yee Lo

**Affiliations:** 1 The University of Hong Kong, Pokfulam, Hong Kong, Special Administrative Region of China; 2 The Chinese University of Hong Kong, Shatin, Hong Kong, Special Administrative Region of China

**Keywords:** Item response theory, Outcome score linking, Depressive symptomatology, Measurement precision, Score concordances, Patient-reported outcome measures

## Abstract

**Background:**

Item response theory (IRT) has been increasingly applied to patient-reported outcome (PRO) measures. The purpose of this study is to apply IRT to examine item properties (discrimination and severity of depressive symptoms), measurement precision and score comparability across five depression measures, which is the first study of its kind in the Chinese context.

**Methods:**

A clinical sample of 207 Hong Kong Chinese outpatients was recruited. Data analyses were performed including classical item analysis, IRT concurrent calibration and IRT true score equating. The IRT assumptions of unidimensionality and local independence were tested respectively using confirmatory factor analysis and chi-square statistics. The IRT linking assumptions of construct similarity, equity and subgroup invariance were also tested. The graded response model was applied to concurrently calibrate all five depression measures in a single IRT run, resulting in the item parameter estimates of these measures being placed onto a single common metric. IRT true score equating was implemented to perform the outcome score linking and construct score concordances so as to link scores from one measure to corresponding scores on another measure for direct comparability.

**Results:**

Findings suggested that (a) symptoms on depressed mood, suicidality and feeling of worthlessness served as the strongest discriminating indicators, and symptoms concerning suicidality, changes in appetite, depressed mood, feeling of worthlessness and psychomotor agitation or retardation reflected high levels of severity in the clinical sample. (b) The five depression measures contributed to various degrees of measurement precision at varied levels of depression. (c) After outcome score linking was performed across the five measures, the cut-off scores led to either consistent or discrepant diagnoses for depression.

**Conclusions:**

The study provides additional evidence regarding the psychometric properties and clinical utility of the five depression measures, offers methodological contributions to the appropriate use of IRT in PRO measures, and helps elucidate cultural variation in depressive symptomatology. The approach of concurrently calibrating and linking multiple PRO measures can be applied to the assessment of PROs other than the depression context.

## Background

With a growing emphasis on patient-centered care, the recent surge in the use of high-quality data from psychometrically sound patient-reported outcome (PRO) measures has engendered the opportunity to use PROs to inform healthcare practices and guide healthcare decision making. In a commissioned paper by the U.S. National Quality Forum on the issues to consider when evaluating PROs as candidate performance measures in healthcare settings, Cella et al. [1] remarked on several methodological issues related to the use of PROs in patient-centered outcome research. One issue focuses on establishing standardized metrics and deriving comparable scores across different PRO measures of the same construct to facilitate direct comparisons between PROs. In addition, the authors highlighted a number of PRO characteristics to consider when selecting appropriate PROs. Measurement precision was among the most important characteristics, as PRO measures with greater measurement precision appear to show greater sensitivity to change [1]. PRO measures not only have great potential to be integrated into healthcare practice but also substantially contribute to elucidating the properties of symptoms directly reported by patients (see for example [2]).

In response to the aforementioned methodological issues, item response theory (IRT) [3] offers promising solutions to address issues that have been difficult to solve through classical methods, and recently, IRT has been increasingly applied to PRO measures. In comparison with classical test theory, IRT offers a number of benefits. First, the application of IRT in examinations of item properties (items can be considered symptoms) adds knowledge regarding the level of severity and discriminating abilities of various symptoms. Such knowledge is of particular clinical interest for assessing symptomatology, as some items may hold higher discriminatory power for differentiating varied levels of clinical latent traits, while other items may reflect more severe symptoms. Second, comparisons from IRT-derived test information functions and their associated standard errors of measurement yield useful information about the contribution of different measures to measurement precision along the latent trait continuum. Clinicians can then determine the most useful and precise measures for assessing a specific level/range of the latent trait in either clinical or epidemiological populations. Third, IRT allows for a common metric on which the item parameters of multiple measures can be placed, and hence, score concordances can be constructed to link scores from one measure to corresponding scores on another measure, in order to facilitate direct comparability across measures. Clinicians can then further investigate whether the conventional cut-off scores on different measures lead to a convergent or divergent solution for clinical and epidemiological decision making.

Major depressive disorder (MDD) is among the significant causes of disease burden worldwide [4]. Regarding the measures of depressive symptomatology, to date, several well developed and carefully validated PRO measures, such as the Beck Depression Inventory–II (BDI-II) [5], the Center for Epidemiologic Studies Depression Scale (CES-D) [6], the Patient Health Questionnaire (PHQ-9) [7], the depression subscale of the Depression, Anxiety and Stress Scale (DASS-Depression) [8], and the depression subscale of the Hospital Anxiety and Depression Scale (HADS-Depression) [9], have been widely used in research and clinical practice. These instruments have been validated in the Chinese context with proven evidence of sound reliability and validity based primarily on classical test theory [10–18]. Under the IRT framework, studies conducted exclusively in Western cultures have offered good examples of comparing and linking multiple depression measures [19–25]. However, considering the existence of cultural variance in the assessments of depression [26–28], whether the aforementioned findings developed in Western populations can be applicable to the Chinese context remains unclear. Cultural differences in terms of item endorsement in these commonly used depression inventories had been noted in past studies [26, 29]. Dere et al. [26] for example noted that Canadian university students of Chinese heritage tended to score higher on cognitive items (e.g., past failure, worthlessness) than their European-heritage counterparts in BDI-II. However, the aforementioned studies were conducted by comparing Caucasian-heritage and Asian-heritage students and it remains unknown whether the findings could be generalized to native Chinese samples, particularly among clinically depressed samples. In addition, no studies thus far have attempted to apply IRT, a modern measurement technique, to multiple depression measures by examining item properties, measurement precision and score comparability together in the Chinese context.

Therefore, the present study attempts to fill this gap by applying IRT to measure depression through an examination of five depression measures (i.e., the BDI-II, CES-D, PHQ-9, DASS-Depression and HADS-Depression) in a clinical sample of depressed Chinese adults. Specifically, the following questions are addressed: (a) What levels of severity and discrimination are associated with the individual depressive symptoms assessed by the five measures? (b) To what extent can each of the five measures contribute to measurement precision in assessments of a full range of underlying depression levels? (c) What is the relationship between the scores from one measure and the corresponding scores from another measure? A clinical sample (*N* = 207) of Hong Kong Chinese outpatients seeking treatment for mood and anxiety disorders was recruited from local hospitals for this study.

## Methods

### Sample

In the original sample, 207 Hong Kong Chinese outpatients seeking treatment for mood and anxiety disorders in Hong Kong public hospitals were invited to participate in the study. Those who were suffering from psychotic or developmental disorders at the time of testing were excluded. The sample comprised 42 males (20.3%) and 165 females (79.7%) ranging in age from 19 to 69 years (*M* = 45.7 years, *SD* = 10.8). Detailed sample characteristics are reported in Table 1. Among the 207 respondents, all participants (100%) completed the BDI-II, the DASS-Depression, and the HADS-Depression, 204 out of the 207 respondents (98.6%) completed the PHQ-9, and 199 out of the 207 respondents (96.1%) completed the CES-D. No data on the completed measures were missing.

**Table 1:** Sample Characteristics (*N* = 207)

	Frequency (*f*)	Percentage (%)
Gender		
Female	165	20.3
Male	42	79.7
Age		
19 − 29	20	9.7
30 − 39	37	17.9
40 − 49	63	30.4
50 − 59	74	35.7
60 − 69	13	6.3
Marital status		
Married	97	46.9
Widowed	22	10.6
Divorced	45	21.7
Separated	5	2.4
Single	37	17.9
NA	1	0.5
Education		
Primary	41	19.8
Secondary	140	67.6
Tertiary	26	12.6
Diagnoses^a^		
Major Depressive Disorder Only	59	28.5
Major Depressive Disorder with Comorbid Conditions (e.g., Anxiety Disorders)	84	40.6
Dysthymia Only	9	4.3
Dysthymia with Comorbid Conditions	9	4.3
Other Conditions (e.g., Bipolar Disorder, Mood Disorders due to General Medical Conditions)	46	22.3

Note

^a^Depression was diagnosed by the Structured Clinical Interview for the Diagnostic and Statistical Manual of Mental Disorders-Fourth Edition (SCID) [33]

### Measures (Diagnostic interview and self-report questionnaires including the BDI-II, CES-D, PHQ-9, DASS, and HADS)

The Structured Clinical Interview for DSM-IV-TR Axis I Disorders (SCID) [30] was administered to screen depressed patients. The 21-item BDI-II [5] was designed to assess cognitive, behavioral and somatic symptoms of depression. The CES-D is a 20-item measure designed to assess depressive symptoms in epidemiological studies focusing on the affective component of depression [6, 31, 32]. As a screening and diagnostic tool, the PHQ-9 is a nine-item instrument designed for use in primary care [7], on the basis of the criteria for MDD in the Diagnostic and Statistical Manual of Mental Disorders-Fourth Edition (DSM–IV) [33]. The 21-item DASS was designed to measure three related negative emotional states–depression, anxiety and tension/stress [8]. The HADS was developed to assess anxiety and depression in medical patients [9] with the exclusion of somatic symptoms (e.g., sleep disturbance) in order to avoid confounding psychological symptoms with disease or treatment. The Chinese versions of these measures were demonstrated sound reliability and validity for use with Chinese populations [10, 11, 13–16, 18].

### Procedure

Participants were tested individually upon providing written consent. They were invited to complete the SCID and a series of self-report depression and anxiety measurement instruments. Ethics approval was obtained from the Joint Institutional Review Board of the University of Hong Kong – Hospital Authority Hong Kong West Cluster and the Joint Chinese University of Hong Kong – New Territories East Cluster Clinical Research Ethics Committee.

### Statistical analysis

#### Classical item analysis

Prior to fitting the IRT model, we performed classical item analysis to examine the item quality and determine the IRT model selection. At the item level, frequencies for each response category (ranging from 0 to 3), means, standard deviations and item total correlations were computed. At the scale level, means and standard deviations of observed summed scores and Cronbach’s alpha values were calculated. Items with a broad range of item total correlations indicate the need for a discrimination parameter when an IRT model is selected.

#### IRT assumption checking

We tested two IRT assumptions: unidimensionality and local independence. To determine essential unidimensionality, a value of 4 for the ratio of the first to the second eigenvalues is generally accepted to support unidimensionality [34]. Further, a single-factor confirmatory factor analysis (CFA) model was employed based on polychoric correlations with a weighted least squares estimation using Mplus 6 [35]. A single-factor CFA model was run on each measure independently to provide evidence of validity based on the internal structure. As we planned our IRT concurrent calibration on the combined item set comprising all five measures, we performed a CFA on the combined dataset. A good fit of the single-factor solution supports the unidimensionality assumption. Adequate fit is generally indicated by a comparative fit index (CFI) value above .90, a Tucker Lewis index (TLI) value above .90, and a root mean square error of approximation (RMSEA) value below .10, while very good fit is typically indicated by a CFI value above .95, a TLI value above .95 and a RMSEA value below .05 [36–39].

Next, we assessed local dependence between item pairs by using Chen and Thissen’s chi-square local dependence statistics (LD *χ* ^2^) [40] in IRTPRO [41]. An LD *χ* ^2^ value of 10 or greater [40, 42] indicate local dependencies.

#### IRT concurrent calibration and goodness-of-fit assessment

The combined item set comprising the five measures was concurrently calibrated in a single IRT run by using the graded response model (GRM) [43] in MULTILOG7.03 [44] so that the item parameter estimates were placed onto a single common metric. Further, we checked the standard errors (*SE*s) of the item parameter estimates to ensure that the GRM was well estimated. Average *SE* values for item parameters between .20 and .35 indicate good estimates [45]. Additionally, we evaluated the degree of fit between the IRT model and the data by using Orlando and Thissen’s summed-score item-fit statistics (*S-X* ^2^) [46]. A nonsignificant result indicates adequate model fit.

#### Outcome score linking and score concordances construction

Linking secures the comparability of scores across different measures and typically consists of three steps: (a) selecting a data collection design, (b) placing parameter estimates on a common metric, and (c) linking test scores. A single-group design in which each respondent was administered all five depression instruments was adopted. Concurrent calibration was performed to place parameter estimates on a common metric. IRT true score equating [47] was implemented in POLYEQUATE [48] to perform the outcome score linking and construct score concordances in order to transfer every possible summed score to a corresponding IRT-derived θ score and associate the summed scores across the five measures. Before performing the linking, we tested the linking assumptions of construct similarity, equity and subgroup invariance [49].

## Results

### Classical item analysis

The wide range of observed summed scores for each measure (Table 2) ensured good coverage of the whole spectrum of depression levels ranging from low to high. Cronbach’s alpha values (ranging from .82 to .92) across the five measures and the overall alpha for the combined item set (α = .98) indicated high reliability. The variety of item total correlations on the combined item set (ranging from .21 to .81) suggested that an IRT model accounting for the heterogeneity in discrimination parameters was necessary.

**Table 2:** Results from classical item analysis and unidimensionality analysis of BDI-II, CES-D, PHQ-9, DASS-Depression, and HADS-Depression

Scale^a^	*no* ^b^	*M* ^c^	*SD* ^d^	α^e^	Item-Total Correlation		Exploratory Factor Analysis (EFA)			Confirmatory Factor Analysis (CFA)		
					Range	Mean	First eigenvalue	Second eigenvalue	Ratio^f^	CFI^g^	TLI^h^	RMSEA^i^
BDI-II	21	22.4	13.0	.92	.43 – .73	.59	9.49	1.10	8.63	0.95	0.94	0.075
CES-D	20	26.2	11.9	.92	.27 – .79	.59	9.30	1.04	8.94	0.94	0.93	0.093
PHQ-9	9	10.8	7.2	.91	.61 – .79	.68	5.59	0.37	15.11	0.97	0.97	0.117
DASS-Depression	7	15.5	11.2	.91	.58 – .80	.73	4.81	0.25	19.24	0.98	0.98	0.119
HADS-Depression	7	8.7	4.5	.82	.44 – .65	.57	3.42	0.29	11.79	0.98	0.97	0.093
All Items	64	-	-	.98	.21 – .81	.62	29.95	2.52	11.88	0.95	0.95	0.051
		Correlation (in lower triangle)/Disattentuated Correlations (in upper triangle)										
		BDI-II			CES-D		PHQ-9		DASS-Depression		HADS-Depression	
BDI-II		*-*			0.91		0.88		0.88		0.86	
CES-D		0.84			*-*		0.93		0.91		0.86	
PHQ-9		0.81			0.85		*-*		0.89		0.85	
DASS-Depression		0.81			0.83		0.81		*-*		0.87	
HADS-Depression		0.75			0.75		0.73		0.75		-	

Note

^a^ BDI-II = Beck Depression Inventory–II. Raw scores range from 0 to 63; higher scores indicate more depressive symptoms

CES-D = Center for Epidemiologic Studies Depression Scale. Raw scores range from 0 to 60; higher scores indicate more depressive symptoms

PHQ-9 = Nine-item Patient Health Questionnaire. Raw scores range from 0 to 27; higher scores indicate more depressive symptoms

DASS-Depression = Depression subscale of the 21-item Depression, Anxiety and Stress Scale. Raw scores range from 0 to 21; higher scores indicate more depressive symptoms

HADS-Depression = Depression subscale of the Hospital Anxiety and Depression Scale. Raw scores range from 0 to 21; higher scores indicate more depressive symptoms

^b^ *no* = number of items

^c^ *M* = mean

^d^ *SD* = standard deviation

^e^ α = Cronbach’s alpha

^f^ Ratio = the ratio of the first eigenvalue to the second eigenvalue (Ratio > 4 supports unidimensionality)

^g^ CFI = comparative fit index (CFI > .90 indicates adequate fit; CFI > .95 indicates very good fit)

^h^ TLI = Tucker Lewis index (TLI > .90 indicates adequate fit; TLI > .95 indicates very good fit)

^i^ RMSEA = root mean square error of approximation (RMSEA < .10 indicates adequate fit; RMSEA < .05 indicates very good fit)

### IRT assumption checking

For each depression measure and the combined item set, the ratio of the first to the second eigenvalues considerably exceeded 4. From the CFA, the fit statistics suggested either adequate or very good fit depending on the fit statistics referenced (Table 2). Notably, for the combined item set for which the IRT calibration was planned, the fit statistics showed very good fit (CFI = 0.95, RMSEA = 0.051, TLI = 0.95). All these results lend support to the essential unidimensional assumption.

Local independence was largely assumed, with the exception of one item pair. Between BDI-II item “Crying” and CES-D item “I had crying spells”, this item pair exhibited a LD *χ* ^2^ value slightly higher than 10 (*χ* ^2^ = 10.4), likely because the items were similar in content.

Considering that the data were essentially unidimensional and that almost all item pairs were locally independent, we considered that the data were suitable for IRT calibration and thus proceeded with the IRT analysis.

### Evaluation of linking assumptions

The linking assumptions of construct similarity, equity and subgroup invariance were tested for the appropriateness of linking. To ensure that the five scales essentially measure the same or similar underlying constructs, we considered the single factor solution from the CFA and the high level of internal consistency from Cronbach’s alpha on the combined item set (*α* = .98) to be supporting evidence of construct similarity. To ensure that the scores of the five measures to be linked were highly correlated for the equity assumption, we computed correlations (ranging from .73 to .85) and disattenuated correlations (ranging from .85 to .93) in the pairwise observed scale scores (Table 2), indicating that the five measures were strongly correlated. In terms of the subgroup invariance assumption, the same item function relating IRT-derived θ scores and summed scores generally held across gender groups, providing support for the subgroup invariance assumption.

### IRT concurrent calibration and goodness-of-fit assessment

#### Evaluation of estimation accuracy and model-data fit

Although the sample was of moderate size (207 participants), the average *SE*s for item parameters ranged between .20 and .30 (Table 3), demonstrating that the IRT model was well estimated. It suggested that acceptable estimation accuracy was largely achieved in this IRT calibration.

**Table 3:** Item content, response frequencies, IRT item parameter estimates and fit statistics

Item^a^	Description^b^	Response Frequencies (%)				Item Parameter Estimates^c^				Fit Index^d^
		0	1	2	3	*a* (*SE*)	*b*_1_ (*SE)*	*b*_2_ (*SE*)	*b*_3_ (*SE*)	*S-X* ^*2*^
BDI_1	Sadness (DM)	52.17	37.20	7.73	2.90	1.94 (0.33)	−0.01 (0.13)	1.53 (0.21)	2.40 (0.35)	76.69^*^
BDI_2	Pessimism (FH)	28.99	26.57	29.47	14.98	1.48 (0.23)	−0.88 (0.19)	0.15 (0.14)	1.47 (0.25)	76.03
BDI_3	Past failure (FH)	37.75	23.04	28.43	10.78	1.59 (0.26)	−0.54 (0.17)	0.33 (0.14)	1.71 (0.27)	69.55
BDI_4	Loss of pleasure (LI)	34.47	40.78	17.96	6.80	2.20 (0.32)	−0.59 (0.13)	0.80 (0.13)	1.79 (0.22)	52.11
BDI_5	Guilty feelings (FH)	38.83	36.41	18.45	6.31	1.31 (0.25)	−0.57 (0.2)	0.97 (0.21)	2.40 (0.43)	99.76^*^
BDI_6	Punishment feelings (FH)	42.23	24.27	7.28	26.21	1.42 (0.27)	−0.40 (0.17)	0.56 (0.18)	0.89 (0.21)	83.45^*^
BDI_7	Self-dislike (FH)	41.26	27.67	20.39	10.68	1.73 (0.27)	−0.36 (0.15)	0.67 (0.14)	1.67 (0.26)	80.58
BDI_8	Self-criticalness (FH)	33.50	28.16	25.24	13.11	1.27 (0.23)	−0.79 (0.21)	0.46 (0.19)	1.82 (0.33)	77.64
BDI_9	Suicidal thoughts (SU)	48.54	37.38	7.28	6.80	1.74 (0.29)	−0.16 (0.13)	1.41 (0.21)	2.02 (0.29)	71.33
BDI_10	Crying (DM)	42.51	23.19	11.59	22.71	1.12 (0.21)	−0.37 (0.22)	0.72 (0.22)	1.31 (0.30)	87.00
BDI_11	Agitation (PA)	45.41	25.12	17.39	12.08	1.36 (0.25)	−0.22 (0.19)	0.88 (0.21)	1.85 (0.37)	72.99
BDI_12	Loss of interest (LI)	30.43	36.23	20.29	13.04	1.88 (0.25)	−0.78 (0.14)	0.55 (0.15)	1.49 (0.21)	64.79
BDI_13	Indecisiveness (CD)	32.52	38.35	24.27	4.85	1.58 (0.25)	−0.75 (0.18)	0.76 (0.17)	2.39 (0.41)	59.79
BDI_14	Worthlessness (FH)	41.55	28.99	15.94	13.53	2.17 (0.30)	−0.37 (0.12)	0.62 (0.13)	1.33 (0.19)	76.83
BDI_15	Loss of energy (LE)	15.94	43.00	30.92	10.14	1.58 (0.22)	−1.62 (0.25)	0.24 (0.15)	1.77 (0.26)	74.41
BDI_16	Changes in sleep (CS)	15.20	42.16	23.04	19.61	0.84 (0.18)	−2.46 (0.56)	0.27 (0.26)	1.80 (0.43)	92.60
BDI_17	Irritability (DM)	40.58	37.68	16.43	5.31	1.27 (0.22)	−0.52 (0.18)	1.19 (0.25)	2.68 (0.49)	59.40
BDI_18	Changes in appetite (WC)	44.17	33.98	15.53	6.31	1.01 (0.21)	−0.41 (0.21)	1.39 (0.34)	2.99 (0.66)	76.75
BDI_19	Concentration difficulty (CD)	31.55	33.98	27.18	7.28	1.45 (0.26)	−0.84 (0.19)	0.54 (0.18)	2.20 (0.37)	74.45
BDI_20	Tiredness or fatigue (LE)	17.87	50.72	25.12	6.28	1.50 (0.24)	−1.51 (0.25)	0.66 (0.18)	2.26 (0.36)	63.96
BDI_21	Loss of interest in sex (LI)	29.90	24.02	21.57	24.51	1.17 (0.20)	−1.02 (0.24)	0.11 (0.2)	1.16 (0.25)	92.37
CESD_1	Bothered by things (CD)	26.57	49.76	16.91	6.76	1.54 (0.26)	−1.03 (0.20)	1.00 (0.19)	2.16 (0.36)	70.99
CESD_2	My appetite was poor (WC)	54.59	33.82	7.25	4.35	1.28 (0.26)	0.10 (0.17)	1.91 (0.37)	2.84 (0.58)	61.61
CESD_3	Couldn’t shake off blues (DM)	28.02	34.78	21.26	15.94	2.08 (0.26)	−0.86 (0.14)	0.37 (0.12)	1.22 (0.16)	74.36
CESD_4	I am just as good as other people (FH)	5.85	8.78	39.02	46.34	0.36 (0.17)	−7.85 (3.14)	−4.98 (2.09)	0.38 (0.61)	108.74^*^
CESD_5	I had trouble concentrating (CD)	27.05	43.48	23.19	6.28	1.19 (0.21)	−1.18 (0.25)	0.82 (0.21)	2.61 (0.48)	75.92
CESD_6	I felt depressed (DM)	21.36	40.78	24.27	13.59	2.89 (0.41)	−1.03 (0.13)	0.31 (0.09)	1.20 (0.14)	65.88
CESD_7	Everything I did was an effort (LE)	25.12	42.51	22.71	9.66	1.92 (0.27)	−1.00 (0.17)	0.55 (0.13)	1.64 (0.23)	58.60
CESD_8	I felt good about the future (FH)	5.31	11.11	35.75	47.83	0.77 (0.17)	−4.18 (1.02)	−2.45 (0.55)	0.03 (0.27)	119.10^*^
CESD_9	I thought I was a failure (FH)	30.92	34.30	21.74	13.04	2.33 (0.33)	−0.72 (0.13)	0.40 (0.11)	1.30 (0.17)	73.36
CESD_10	I felt fearful (DM)	30.92	38.65	19.32	11.11	1.62 (0.27)	−0.81 (0.17)	0.67 (0.15)	1.67 (0.26)	88.92
CESD_11	My sleep was restless (CS)	21.84	29.61	23.79	24.76	1.35 (0.21)	−1.36 (0.24)	0.00 (0.17)	1.04 (0.23)	73.10
CESD_12	I was happy (DM)	6.31	15.53	46.60	31.55	1.02 (0.17)	−3.26 (0.63)	−1.61 (0.33)	0.84 (0.26)	102.95^*^
CESD_13	I talked less than usual (PA)	30.92	38.16	19.32	11.59	1.61 (0.22)	−0.82 (0.18)	0.59 (0.15)	1.61 (0.25)	57.09
CESD_14	I felt lonely (DM)	33.33	30.43	18.36	17.87	1.93 (0.25)	−0.70 (0.14)	0.34 (0.13)	1.11 (0.17)	76.70
CESD_15	People were unfriendly (FH)	41.06	43.00	13.04	2.90	1.37 (0.25)	−0.42 (0.18)	1.52 (0.30)	3.01 (0.57)	67.50
CESD_16	I enjoyed life (LI)	6.31	12.14	34.95	46.60	1.35 (0.20)	−2.71 (0.44)	−1.55 (0.26)	0.06 (0.17)	69.02
CESD_17	I had crying spells (DM)	51.69	34.78	8.70	4.83	1.45 (0.27)	−0.04 (0.15)	1.55 (0.25)	2.46 (0.41)	56.71
CESD_18	I felt sad (DM)	35.27	35.27	17.39	12.08	2.96 (0.39)	−0.53 (0.09)	0.55 (0.10)	1.27 (0.14)	51.57
CESD_19	I felt that people disliked me (FH)	39.32	39.32	16.99	4.37	1.49 (0.29)	−0.51 (0.17)	1.12 (0.22)	2.52 (0.44)	59.90
CESD_20	I could not “get going.” (DM)	21.95	40.49	22.93	14.63	2.37 (0.33)	−1.09 (0.14)	0.31 (0.12)	1.24 (0.15)	66.01
PHQ_1	Little interest (LI)	27.54	41.55	18.36	12.56	2.94 (0.37)	−0.79 (0.11)	0.51 (0.10)	1.25 (0.14)	60.36
PHQ_2	Feeling down, depressed, or hopeless (DM)	30.43	37.20	22.22	10.14	3.43 (0.44)	−0.67 (0.09)	0.45 (0.09)	1.35 (0.13)	73.78^*^
PHQ_3	Sleep disturbance (CS)	16.50	34.47	17.48	31.55	1.58 (0.22)	−1.56 (0.22)	−0.01 (0.16)	0.68 (0.17)	82.03
PHQ_4	Feeling tired or having little energy (LE)	17.39	34.30	26.57	21.74	1.73 (0.26)	−1.43 (0.20)	−0.01 (0.13)	1.02 (0.18)	84.14^*^
PHQ_5	Poor appetite or overeating (WC)	45.89	28.99	13.04	12.08	1.40 (0.24)	−0.26 (0.16)	0.94 (0.20)	1.75 (0.31)	74.69
PHQ_6	Feeling bad about oneself (FH)	35.27	29.47	21.26	14.01	2.41 (0.31)	−0.57 (0.11)	0.35 (0.11)	1.23 (0.16)	83.59^*^
PHQ_7	Trouble concentrating (CD)	38.16	33.33	16.91	11.59	1.63 (0.23)	−0.52 (0.16)	0.75 (0.16)	1.64 (0.25)	78.16
PHQ_8	Moving or speaking slowly (PA)	49.76	28.99	13.53	7.73	1.67 (0.28)	−0.13 (0.14)	1.01 (0.19)	1.96 (0.29)	57.67
PHQ_9	Thoughts of death (SU)	67.48	18.45	8.25	5.83	2.76 (0.46)	0.44 (0.09)	1.15 (0.15)	1.74 (0.20)	37.89
DASS_3	No positive feeling at all (DM)	27.67	34.95	24.27	13.11	3.00 (0.37)	−0.79 (0.10)	0.30 (0.10)	1.22 (0.14)	66.74
DASS_5	No initiatives (LI)	27.54	43.48	19.81	9.18	1.45 (0.24)	−1.03 (0.19)	0.78 (0.19)	1.99 (0.34)	81.62
DASS_10	Had nothing to look forward to (FH)	30.43	27.54	22.71	19.32	2.39 (0.31)	−0.71 (0.12)	0.19 (0.11)	0.99 (0.15)	61.77
DASS_13	Felt down-hearted and blue (DM)	21.26	38.65	23.67	16.43	3.11 (0.35)	−1.02 (0.12)	0.20 (0.09)	1.02 (0.13)	49.35
DASS_16	Unable to become enthusiastic (LI)	27.67	44.17	16.50	11.65	1.90 (0.27)	−0.91 (0.14)	0.68 (0.15)	1.54 (0.21)	71.35
DASS_17	Wasn’t worth much as a person (FH)	56.52	26.09	9.66	7.73	2.14 (0.34)	0.11 (0.11)	1.09 (0.16)	1.73 (0.22)	54.99
DASS_21	Life was meaningless (FH)	44.93	27.54	14.98	12.56	2.54 (0.37)	−0.24 (0.10)	0.63 (0.11)	1.29 (0.16)	68.49
HADS_2	Enjoy the things I used to enjoy (LI)	21.26	44.44	27.54	6.76	1.45 (0.23)	−1.32 (0.23)	0.55 (0.16)	2.20 (0.35)	78.75
HADS_4	Laugh and see the funny side of things (LI)	34.30	26.09	29.95	9.66	2.02 (0.26)	−0.59 (0.15)	0.29 (0.12)	1.61 (0.21)	61.69
HADS_6	Feel cheerful (DM)	10.14	50.24	30.92	8.70	1.93 (0.28)	−1.89 (0.21)	0.28 (0.12)	1.69 (0.25)	77.59
HADS_8	Feel as if I am slowed down (PA)	14.49	47.34	27.54	10.63	1.27 (0.22)	−1.94 (0.30)	0.41 (0.19)	2.04 (0.37)	72.17
HADS_10	Lost interest in my appearance (LI)	22.71	41.06	25.60	10.63	0.87 (0.19)	−1.74 (0.41)	0.64 (0.27)	2.65 (0.60)	85.76
HADS_12	Look forward with enjoyment to things (LI)	23.19	31.88	30.43	14.49	1.58 (0.24)	−1.2 (0.20)	0.09 (0.14)	1.47 (0.24)	89.89
HADS_14	Enjoy good book or radio/TV program (LI)	36.23	35.75	14.01	14.01	1.16 (0.21)	−0.71 (0.21)	0.92 (0.22)	1.81 (0.35)	88.73

Note

^*^ *p* < Benjamini-Hochberg adjusted overall alpha level of .05 [69]

^a^ BDI = Beck Depression Inventory–II

CESD = Center for Epidemiologic Studies Depression Scale

PHQ = Nine-item Patient Health Questionnaire

DASS = Depression subscale of the 21-item Depression, Anxiety and Stress Scale

HADS = Depression subscale of the Hospital Anxiety and Depression Scale

^b^ CD = concentration difficulties; CS = change in sleep; DM = depressed mood; FH = feelings of hopelessness; LE = fatigue or loss of energy; LI = loss of interest; PA = psychomotor agitation/retardation; SU = suicidality; WC = significant weight change or change in appetite

^c^ *a* = item discrimination parameter estimates; *b*_1_, *b*_2_, *b*_3_ = item severity parameter estimates; *SE* = standard error of corresponding item parameter estimates

^d^ Fit index: Orlando and Thissen’s summed-score item-fit statistics (*S-X* ^*2*^). A nonsignificant result with Benjamini-Hochberg [69] adjusted overall alpha level of .05 is an indicator of adequate model fit

Nine items were reported to show a lack of fit, while good fit was indicated for the rest of the items (Table 3). We further examined the consequence of item misfit on the item and person parameter estimates and found that either including or excluding the nine items yielded nearly identical results. Therefore, as we considered the consequence minor and the misfit tolerable [50], we included all items in the outcome score linking.

#### Comparison of item properties across the five depression measures

The item discrimination (*a*) parameters (Table 3) across the five measures ranged in value from 0.36 to 3.43 (*M* = 1.73, *SD* = 0.62). Notably, items addressing depressed mood, suicidality and feelings of worthlessness provided the strongest discriminating indicators; thus, they were the most useful for discriminating among respondents with varied levels of depression. The second highly discriminating set of indicators included items on fatigue or loss of energy, psychomotor agitation or retardation, and concentration difficulties. The moderately discriminating set of indicators contained items on changes in sleep and changes in appetite. CES-D items on positive affect (i.e., “I am just as good as other people”, “I felt good about the future” and “I was happy”) had the weakest ability to distinguish respondents with varied depression levels and thus added the least information to the depression measurement. Of additional interest was the great variation in the discriminating abilities of items on loss of interest (*a* parameter estimates ranging from 0.87 to 2.94).

Regarding item severity (*b*) parameters (Table 3), symptoms pertaining to suicidality, changes in appetite, depressed mood, feelings of worthlessness and psychomotor agitation or retardation were associated with high levels of severity. Items on concentration difficulties, fatigue or loss of energy and loss of interest, followed by problems related to changes in sleep, were associated with moderate levels of severity. All of the four CES-D items on positive affect were associated with the lowest levels of severity.

**Fig. 1:**
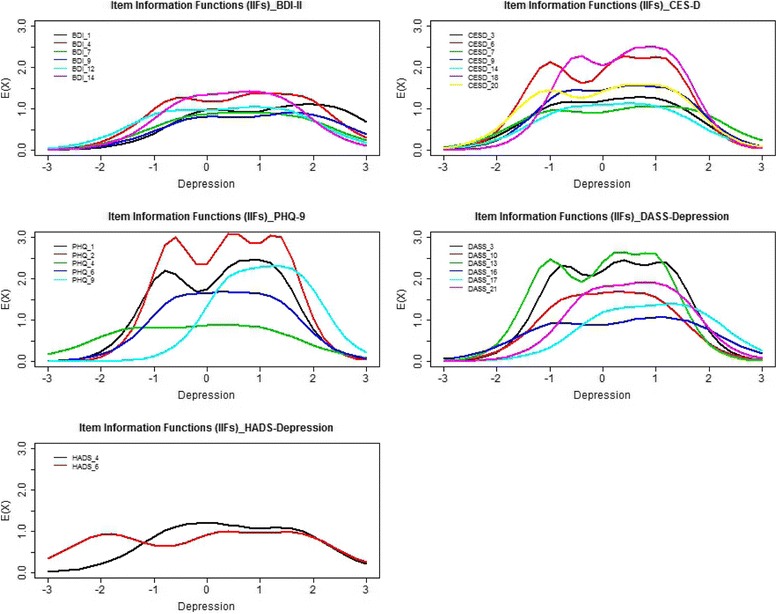
Item information functions (IIFs) Curves. *Note*. Using Baker’s criteria (*a* < 0.64 = very low or low, 0.65 < *a* < 1.34 = moderate, 1.35 < *a* < 1.69 = high, *a* > 1.7 = very high) [70], the item information function curves for items with very high item discrimination parameter estimates (*a* >1.7) were plotted. Such higher discriminating items provide more information with greater precision for measuring a respondent’s level of depression and are thus particularly useful. BDI-II = Beck Depression Inventory–II; CES-D = Center for Epidemiologic Studies Depression Scale; PHQ-9 = 9-item Patient Health Questionnaire; DASS-Depression = Depression subscale of the 21-item Depression, Anxiety and Stress Scale; HADS-Depression = Depression subscale of the Hospital Anxiety and Depression Scale

With respect to item information, among the items with similar *a* values, the level of precision/usefulness for assessing depression differed along the θ continuum. For instance, between DASS-Depression items “Felt down-hearted and blue” (*a* = 3.11) and “No positive feeling at all” (*a* = 3.00), the former was more useful for differentiating respondents with depression levels along θ < −0.8 and 0 < θ <1, and the latter was more informative for discriminating respondents with depression levels along −0.8 < θ < 0 (Fig. 1).

### Outcome score linking and score concordances construction

#### Comparison of cut-off theta scores across the five depression measures

Each (observed) summed score for each measure transferred to an IRT-derived θ (theta) score. The score concordances at cut-off scores are reported in Table 4. For instance, in the 20-item CES-D, a summed score of 16, the cut-off point for identifying respondents as being at risk for depression, transferred to a θ score of −0.95, indicating that the cut-off score of 16 distinguished people with a θ score above −0.95 from those with a θ score below −0.95.

**Table 4:** Score concordances at cut-off scores of BDI-II, CES-D, PHQ-9, DASS-Depression, and HADS-Depression

	Cut-off Scores						
Scale^a^	Summed Score	IRT score (θ)^b^	Corresponding Summed Score in BDI-II	Corresponding Summed Score in CES-D	Corresponding Summed Score in PHQ-9	Corresponding Summed Score in DASS-Depression	Corresponding Summed Score in HADS-Depression
BDI-II							
Mild depression	14	−0.67	-	19	5	4	6
Moderate depression	20	−0.18	-	24	8	7	8
Severe depression	29	0.47	-	32	13	11	11
CES-D							
Risk for depression	16	−0.95	11	-	4	3	5
PHQ-9							
Mild depression	5	−0.76	13	18	-	4	6
Moderate depression	10	0.05	23	27	-	8	9
Moderately severe depression	15	0.68	32	35	-	12	12
Severe depression	20	1.30	41	43	-	16	14
DASS-Depression							
Mild depression	5	−0.49	16	21	6	-	7
Moderate depression	7	−0.18	20	24	8	-	8
Severe depression	11	0.47	29	32	13	-	11
Extreme Severe depression	14	0.95	36	39	17	-	13
HADS-Depression							
Mild depression	8	−0.33	18	22	7	6	-
Moderate depression	11	0.40	28	31	12	10	-
Severe depression	15	1.37	42	44	20	16	-

Note

^a^ BDI-II = Beck Depression Inventory–II. Raw scores range from 0 to 63; higher scores indicate more depressive symptoms

CES-D = Center for Epidemiologic Studies Depression Scale. Raw scores range from 0 to 60; higher scores indicate more depressive symptoms

PHQ-9 = Nine-item Patient Health Questionnaire. Raw scores range from 0 to 27; higher scores indicate more depressive symptoms

DASS-Depression = Depression subscale of the 21-item Depression, Anxiety and Stress Scale. Raw scores range from 0 to 21; higher scores indicate more depressive symptoms

HADS-Depression = Depression subscale of the Hospital Anxiety and Depression Scale. Raw scores range from 0 to 21; higher scores indicate more depressive symptoms

^b^ IRT score (θ): IRT derived scores representing levels of depression ranging from low to high. Higher values indicate higher depression levels

#### Comparison of cut-off summed scores across the five depression measures

In the same score concordances, each cut-off (observed) summed score for each measure was associated with a (observed) summed score for each of the other four measures (Table 4). Notably, the resulting cut-off scores across the five measures led to either a consistent or discrepant diagnosis for depression. For instance, the cut-off scores for mild depression on the BDI-II and the PHQ-9 were equivalent to each other, whereas the cut-off score for moderate depression on the BDI-II corresponded to the cut-off score for mild depression on the HADS-Depression.

#### Comparison of measurement precision across the five depression measures

**Fig. 2:**
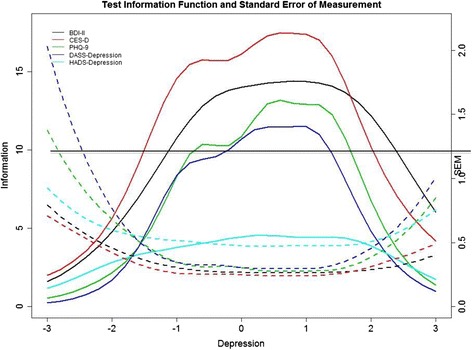
Test information functions (TIFs) and standard errors of measurement (SEMs) Curves. *Note*. Solid lines; left y-axis = total information aggregated across all items within the corresponding scale along the depression level (θ) ranging from −3 to 3. Dashed lines; right y-axis = standard error of measurement at scale level along the depression level (θ) ranging from −3 to 3. The test information of 10 derived from item response theory on the left y-axis is approximately equivalent to the reliability of .90 derived from classical test theory.BDI-II = Beck Depression Inventory–II; CES-D = Center for Epidemiologic Studies Depression Scale; PHQ-9 = 9-item Patient Health Questionnaire; DASS-Depression = Depression subscale of the 21-item Depression, Anxiety and Stress Scale; HADS-Depression = Depression subscale of the Hospital Anxiety and Depression Scale

Concerning the standardization of the five measures’ measurement precision, a test information value of approximately 10 reflects conventional reliability of .90 as derived from classical test theory [51]. As shown in Fig. 2, both the BDI-II and the CES-D were informative on a wider range of depression levels, and they exhibited greater measurement precision than the other three measures, where the BDI-II was more useful for differentiating depression levels for θ scores approximately between −1 and 2.3 (normal to severe depression) and the CES-D was more informative for discriminating respondents with depression levels along θ scores from approximately −1.5 to 2.0. The PHQ-9 offered great potential in assessing depression levels along θ scores from approximately −0.7 to 1.7 (mild to severe depression). The DASS-Depression was informative for assessing depression levels along the θ continuum between −0.3 and 1.3 (moderate to extreme severe depression). Among the five measures, the HADS-Depression was the least informative for assessing varied depression levels, and its maximum test information was roughly equivalent to a conventional reliability of .78.

## Discussion

This is the first study, in the Chinese context, to utilize an IRT approach to the measurement of depression through an examination of five depression measures simultaneously, namely, the BDI-II, CES-D, PHQ-9, DASS-Depression and HADS-Depression.

### Psychometric properties and clinical utility of the five depression measures

The work presented herein significantly contributes to knowledge on depression measurement in the Chinese context. First, the findings from this study demonstrated that the five depression measures had sound reliability and validity for depressed Chinese adults. Our findings join previous studies [10, 11, 13–16, 18, 52–54] in providing supporting evidence of the psychometric properties of these instruments in the same context. Noticeably, CES-D reversely scored items measuring positive affect (e.g., “I am just as good as other people”, “I felt good about the future”) were found to be the least discriminating and to reflect the least severe symptoms; thus, they added little to the measurement precision of depression assessments in the studied context. Our findings echo the work of Iwata et al. [55], who suggested that the CES-D positive affect items with positive wording cannot adequately assess depressive disorders in the Japanese population.

This observation across cultures leads one to rethink more broadly about the role of these instruments in guiding treatment decisions. In determining treatment outcomes, remission is traditionally defined by substantial (or complete) alleviation of depressive symptoms. In the absence of apparent biological state markers for major depression, monitoring of recovery progress could only be defined phenomenologically, often times by comparing patients’ symptom severity with a predetermined diagnostic threshold or clinical cutoff scores in these well-validated depressive inventories [56]. These conceptions, however, were challenged by recent researches advocating a broadening of the concept of remission beyond symptom resolution (e.g., [57, 58]). The new proposal concerns that multiple domains, including for example subjectively perceived functional improvement and quality of life, should also be taken into account if a holistic, patient-centered metric of recovery is considered. In light of this, more comprehensive depression instruments, such as, the Remission from Depression Questionnaire (RDQ), had been developed [59]. From a culturally sensitive perspective, the importance of incorporating these person-centered instruments in addition to standardized depression symptom severity scales were implicated by the present findings, especially when the information is to be used in guiding treatment decisions in the practical field. This is because the benchmark of specific item endorsement on a symptom severity scale may be culturally-dependent, and patients’ perspective on remission status may provide collateral information in helping with efficacious treatment planning tailor made to individual’s needs.

Second, our findings help elucidate cultural variations in depressive symptomatology. Symptoms pertaining to psychologization, such as depressed mood, suicidality and feelings of worthlessness, served as the strongest discriminating indicators, while symptoms pertaining to somatization, such as psychomotor agitation, fatigue or loss of energy, concentration difficulties, changes in sleep and changes in appetite, were found to exhibit highly or moderately discriminating abilities. In terms of severity, symptoms related to suicidality, changes in appetite, depressed mood, and feelings of worthlessness appeared to reflect a high level of severity in the Chinese clinical sample. The findings of the present study share some consistencies with those from previous studies. For instance, suicidality and changes in appetite also emerged at a high level of severity in Western contexts [2, 60]. However, discrepancies do exist. The symptom of feelings of worthlessness was ranked at a relatively low level of severity in the Western context [2], while the same symptom appeared to be rated at a relatively high level of severity by the Chinese outpatients in our study. Similarly, the high level of severity and high discriminating ability of feelings of worthlessness in our findings are in accordance with Saito et al.’s work conducted on a Japanese community sample [61]. Intriguingly, recent work also showed that the cognitive component of negative self-evaluation is an important factor that differentiates reports of depressive symptomatology between Asian and Western youths [62]. The salience of a heightened sense of self-worth may be related to a deep-rooted Confucian value among Asian Chinese, where a person’s intrinsic value is highly dependent on how well the person meets social expectations in serving the collective interest of the social group. Furthermore, a loss of functioning resulting from depression, especially in its severe form, may bring about intense shameful feelings and self-doubt, which further exacerbates a negative vicious cycle of affective-cognitive disruptions.

Intriguingly, a closely related observation is that several items that demonstrated misfit seemed to be associated with a systematic symptom theme. For example, the items “Guilty feeling” and “Punishment feeling” in BDI-II; the items “I am just as good as other people” and “I felt good about the future” in CES-D; and the item “Feeling bad about oneself” in PHQ-9, all loaded onto the same “Feelings of hopelessness” (FH) theme. These items reflect a strong sense of responsibility and echo with the cultural belief that a person’s value should be closely linked with the social roles that one is expected to perform in collectivistic societies. It would be interesting to test if the same pattern of misfitting items would be observed in individualistic cultures in future studies.

Third, the findings on the item and test information offer valuable information regarding how each item/symptom and each measure reliably/precisely assess depression at varied levels. Though they may share similarities in discrimination parameters, items may vary in precision/usefulness for assessing varied levels of depression. For instance, between two DASS-Depression items with similar *a* values, “No positive feeling at all” was more useful for assessing mild and moderate depression, whereas “Felt down-hearted and blue” was more informative for assessing moderate to extreme severe depression. The finding in this example helps us better understand the gradient of affective dysregulation experienced by sufferers of depression and suggests that a loss of positive affect may precede, or interactively exacerbate, the experience of intensive negative affect in the course of depression.

At the scale level, the findings showed that the five depression measures contributed in various degrees to measurement precision along the full range of the underlying depression levels, providing insight into instrument selection. Specifically, in the studied context, the BDI-II and the CES-D were informative on a wider range of depression levels and had greater measurement precision than the other three measures. The PHQ-9 and the DASS-Depression were particularly useful for assessing depression in clinical populations, as the former was informative for measuring depression ranging from mild to severe and latter was informative for assessing depression ranging from moderate to extreme severe. Accordingly, clinicians can choose the measure that is the most useful/precise for assessing a specific level of depressive severity at the patient level in either clinical or epidemiological populations. Notably, the HADS-Depression appeared to be the least informative for assessing depression in the Chinese context, based on the observation that moderate or low discrimination parameter estimates were reported on the majority of items in this scale.

Our pattern of score concordances results echoes previous studies in suggesting that commonly used depression scales seemed to differ in their diagnoses for depression severity. Zimmerman and colleagues [63, 64], for example, administered Hamilton Depression Rating Scale (HRDS), PHQ-9, as well as Clinically Useful Depression Outcome Scale (CUDOS) and Quick Inventory of Depressive Symptomatology (QIDS), to a group of clinically depressed patients and compared the diagnostic outcomes as indicated by the reported scores in each case. The authors noted significant variance in the distribution of patients being classified into discrete levels of severity categories when different scales were used. The level of disagreement implied that treatment planning solely based on data collected from a single self-report scale may be over-inclusive, despite that these scales were all well-validated and standardized.

Finally, the clinical values of the score concordances reported herein are worth highlighting following from the previous point. With scores obtained from the administration of one depression measure, one can use the concordance table to locate the corresponding scores on other depression measures without administering them. Clinicians can then determine depression diagnoses for individual respondents on the basis of the cut-off scores for these rating scales and other interview-based assessments. Further, scores across the five measures are not only aligned with each other in the observed score metric but also mapped to the IRT scores at the θ metric. Such mapping offers clinical meanings for the arbitrary θ metric. For instance, respondents who scored 0.47 or above (at θ metric) on the BDI-II are likely to be diagnosed as severely depressed. Clinicians can then refer to the item information function curves to locate the symptoms that are more informative for assessing this restricted range of severe depression.

### Advantages of the methodology

The methodology used in this in study has several remarkable strengths. First, we followed a single-group design for the outcome score linking. Such a design directly controls for differences in response propensities because the instruments are administered to the same respondents [48]. Additionally, we used concurrent calibration, which is less time-consuming and produces more stable results than separate calibration [48]. Second, we tested the linking assumptions. Such a practice deserves more attention, and it is strongly encouraged in studies on linking PRO measures to ensure the validity of the inferences drawn from the score concordances. Finally, instead of relying solely on chi-square-like IRT fit statistics, which can be sensitive to sample size, we evaluated IRT item misfit by focusing on the consequences of using misfitting items and item statistics associated with them, a strategy strongly recommended by Hambleton and Han [65] and Zhao [50]. We hope that future studies adopting a rigorous approach to addressing methodological issues are encouraged in order to promote the quality of PRO research and to ensure the appropriate application of IRT models.

### Limitations and future directions

The major limitations and future directions of the present study are discussed below. First, a convenient sampling approach was used to recruit participants because of practical restrictions, which limits the representativeness of the sample and the generalizability of the results. A related issue is the unbalanced gender ratio, which limits the power of using statistical tests to examine gender differences. Second, the outcome score linking function/relationship established in the study may be sensitive to population differences [49], and only one linking approach was used in this study. It would seem prudent to evaluate the robustness of the linking relationship across different samples (e.g., in Chinese nonclinical samples) and across multiple linking approaches (e.g., both IRT-based and non-IRT-based approaches). Additionally, whether the invariance of item parameters holds across clinical and nonclinical populations also requires further investigation. With additional sets of larger clinical and epidemiological samples, a more robust item bank and score concordances can be established. Third, the present study did not incorporate other patient-centered instruments in assessing perceived remission status for comparison purpose. As mentioned previously, these patient-centered instruments were informative in defining depression remission with reference to symptom severity and it would be useful to take into account, as well as to explore the potential merits of, these instruments. Future studies could consider including the Remission from Depression Questionnaire [58, 66] and/or the Remission Evaluation and Mood Inventory Tool [67, 68] as examples. Furthermore, it would be useful to conduct follow-up studies with large samples to cross-reference the depression scales with interview-based clinical diagnostic tools relating to depressive symptomatology. Finally, the five depression measures covered in the study have all been developed in Western cultures, although the Chinese versions of these measures have been demonstrated to have sound psychometric properties. Nonetheless, the cut-off scores for depression diagnosis that have been suggested based on the Western context deserve further validation in the Eastern context.

## Conclusions

Based on an examination of five depression measures, the findings of the present study demonstrated (a) levels of severity and discrimination for individual depressive symptoms, (b) measurement precision for each measure at varied levels of depression, and (c) the comparability of severity cut-off scores across the five measures. The study provides additional evidence regarding the psychometric properties and clinical utility of the PRO measures, offers methodological contributions to the appropriate use of IRT models in PRO measures, and, more importantly, enhances our understanding of cultural variation and depressive symptomatology.

## Abbreviations

BDI: Beck depression inventory
CES-D: Center for epidemiologic studies depression scale
CFA: Confirmatory factor analysis
CFI: Comparative fit index
DASS: Depression, anxiety and stress scale
GRM: Graded response model
HADS: Hospital anxiety and depression scale
IRT: Item response theory
LD: Local dependence
MDD: Major depressive disorder
PHQ: Patient Health Questionnaire
PRO: Patient-reported outcome
RMSEA: Root mean square error of approximation
SCID: Structured clinical interview
SE: Standard error
TLI: Tucker Lewis index
